# The Perils of Partnership: Interactions Between Public Health England, Drinkaware, and the Portman Group Surrounding the Drink Free Days Campaign

**DOI:** 10.34172/ijhpm.2024.8245

**Published:** 2024-04-09

**Authors:** Nason Maani, May CI van Schalkwyk, Mark Petticrew

**Affiliations:** ^1^Global Health Policy Unit, School of Social and Political Science, University of Edinburgh, Edinburgh, UK.; ^2^Department of Health Services Research and Policy, Faculty of Public Health and Policy, London School of Hygiene and Tropical Medicine, London, UK.; ^3^Department of Public Health, Environments and Society, Faculty of Public Health and Policy, London School of Hygiene and Tropical Medicine, London, UK.; ^4^UK PRP SPECTRUM Consortium, London School of Hygiene and Tropical Medicine, London, UK.

**Keywords:** Alcohol, Commercial Determinants of Health, Health Policy, Partnership, Public Health, UK

## Abstract

**Background:** There is growing evidence that the alcohol industry seeks to obstruct public health policies that might affect future alcohol sales. In parallel, the alcohol industry funds organisations that engage in "responsible drinking" campaigns. Evidence is growing that the content and delivery of such campaigns serves industry, rather than public health interests, yet these organizations continue to be the subject of partnerships with government health departments. This study aimed to examine the nature and potential impacts of such partnerships by analysing the practices of the alcohol industry-funded charity Drinkaware during the establishment of the Drink Free Days campaign.

**Methods:** A case study based on an inductive analysis of documents revealed by freedom of information (FoI) request regarding communications between Drinkaware, Public Health England (PHE), and the Portman Group, in the years running up to, and during, the Drink Free Days campaign, a partnership between alcohol industry-funded charity Drinkware, and PHE.

**Results:** This study reveals a range of less visible, system-level effects of such partnerships for government departments and civil society. The tensions observed, as exhibited by discrepancies between internal and external communications, the emphasis on managing and mitigating the perception of negative consequences, and the links to wider alcohol industry initiatives and bodies, suggest the need for wider considerations of organizational conflicts of interest, and of possible indirect, harmful consequences to policy-making. These include the marginalization of other civil society voices, the displacing of more effective policy options, and strategic alignment with other industry lobbying activities.

**Conclusion:** The findings have implications for how public health practitioners and health organisations might better weigh the potential trade-offs of partnership in the context of health promotion campaigns.

## Background

**Key Messages**

**Implications for policy makers**

- Through emails obtained via freedom of information (FoI) request regarding the Drink Free Days partnership, this study demonstrates how such partnerships serve commercial interests, including through displacing more effective policy options and marginalising civil society voices
- Policy-makers and the public should be aware of the indirect, sometimes hidden effects of such partnerships and the reputational and strategic risks they may pose.
- Formal processes for ascertaining conflicts of interest, that reflect the evidence base on such third-party organisations, are required to safeguard the mission of public health institutions, as well as their relationships with civil society.
- Public health leaders should prioritise partnerships with organisations based on documented expertise and alignment with the goals of public health, to avoid reputational risks and the undermining of policy best-buys.

**Implications for the public**

This study used communications obtained by freedom of information (FoI) request to examine interactions between Public Health England (PHE), Drinkaware (an alcohol charity that receives funding from the alcohol industry), and the Portman Group (an alcohol industry trade group), in the run-up to and during the “Drink Free Days” campaign, a campaign run in partnership between PHE and Drinkaware. The findings show some of the tensions involved, especially in how the actors involved try to respond to public criticism, or the development of policy, and how such partnerships might serve commercial interests, including through marginalising important civil society voices. The public is often not aware that such partnerships, or the organisations involved, are funded by companies that make and sell alcohol. This study helps demonstrate how and why this matters.

The globally consolidated alcohol industry represents a critical commercial determinant of health.^1^ While evidence for the uniquely harmful nature of this industry continues to grow, including in terms of its structural, strategic and historical parallels (and in some cases co-ownership) with the tobacco industry, it does not face the same restrictions in terms of regulation or governance mechanisms, and does not attract the same level of scrutiny by global health actors. This is perhaps most notable in the context of corporate social responsibility and partnerships, with high-profile, controversial examples such as a partnership with the Global Fund (later rescinded) and coordination between industry representatives and the US National Institute on Alcohol Abuse and Alcoholism.^2^ Partnerships of this nature extend to those mediated via third party organisations, including charities in receipt of alcohol industry funding.

 There is a growing body of evidence that alcohol industry-funded information charities serve the commercial interests of the alcohol industry, and exhibit industry-favourable biases in the health information that they disseminate to the public. This includes misinformation about alcohol consumption and cancer,^3^ foetal alcohol syndrome,^4^ and cardiovascular disease.^5^ The information provided by these charities appears to differ in meaningful ways from non-industry funded sources in their language, social media content,^6^ imagery,^7^ and format.^8^ Their messaging includes a greater emphasis on individual responsibility,^9^ the selective foregrounding of less significant health harms, and the underreporting of major health harms, while avoiding discussion of evidence-based policies to reduce alcohol harm, or the role of the alcohol industry in opposing such policies.^6,10^ However, beyond the content disseminated by such organisations, it is also important to understand the wider strategic purposes they serve through their apparent or claimed separation or ‘independence’ from the industry itself and their practices centered around partnerships with non-industry bodies, particularly legitimate health organisations.^11,12^

 The UK Drink Free Days campaign (2018-2019) offered an example of a high-profile partnership between a national-level public health agency and an alcohol industry-funded health information organization, Drinkaware.^13^ The campaign focused on encouraging middle-aged drinkers to have “drink-free” days for part of the week, and focused on weight gain, blood pressure, and cancer.^14^ Drinkaware itself began as a website established in 2004 by the alcohol industry corporate social responsibility body, the Portman Group, who then established the Drinkaware Trust in 2006 as a separate charity following a memorandum of understanding between the Portman Group and a range of UK government agencies.^15^ Drinkaware is funded by voluntary donations from major UK alcohol producers, supermarkets and other alcohol retailers. The Portman Group describes itself as “the social responsibility body and regulator for alcohol labelling, packaging and promotion in the UK” and that it “aims to consistently challenge the industry to deliver high standards of best practice.”^16^ It provides alcohol health information to the public and policy-makers itself, responds to policy consultations on alcohol harms and policies,^17-19^ and produces infographics, videos and press releases on alcohol consumption trends and harm in the United Kingdom.^20,21^ It has therefore been included in previous analyses of alcohol industry funded health information alongside more explicitly health information-oriented organisations such as Drinkaware.^3^

 Such partnerships serve ongoing strategic functions. In response to Government consultations, the Portman Group continues to make reference to Drinkaware and to Drink Free Days as a means by which industry is helping to address alcohol harms. For example, in its response to the Scottish Governments consultation on Minimum Unit Pricing in 2022, it claimed it played a role in “supporting falls in alcohol consumption and harm,” and as part of the “range of activities” it cites in support of this, the group notes that:

 “…*the industry also voluntarily funds the independent alcohol education charity Drinkaware. It provides free advice and resources to people to help cut down their alcohol consumption, such as through the ‘drink free days campaign’ in co-operation with the UK Government*….”^22^

 The Drink Free Days campaign therefore offers an instructive example of how organisations like Drinkaware or the Portman Group interact with government. Examination of such campaigns may aid in understanding the wider system effects of such partnerships, and whose interests are ultimately served by their formation and outputs. A previous analysis examined the Drink Free Days campaign from the perspective of policy actors, finding there was strong opposition to the campaign among many local authority public health actors, with knowledge regarding the involvement of the alcohol industry in health policy being an important factor affecting perceptions of the partnership.^23^ A separate study consisting of stakeholder interviews and a framing analysis of social media accounts and news coverage regarding the campaign, found that the campaign was helpful to alcohol industry actors strategic aims, by promoting industry-favourable understanding of alcohol harms, normalizing partnership in the minds of public health interviewees, allowing for leverage over government initiatives and pretexts for engagement with policy-makers.^14^

 However, there has been no formal critical analysis of how this partnership was formed, and what practices were adopted by the industry-funded organisations involved at the outset and through the partnership. Such insights are key to understanding and explaining how and why commercial actors may benefit from these partnerships and, importantly, if and how they undermine public goals. This study sought to contribute to this understanding through a case study of the Drink Free Days campaign and the practices of the actors involved in the partnership, namely: Drinkaware, Public Health England (PHE), and the Portman Group, using data obtained by freedom of information (FoI) request. The aim of this case study was to describe the relationships created by the partnership; evaluate the costs and benefits to partners, the mechanisms through which these were achieved; the wider context within which this occurred; and any adverse effects.

## Methods

###  Design

 We conducted a critical analysis of the formation and effects of the partnership that culminated in the delivery of the Drink Free Days campaign using a case study approach,^24^ consistent with previous studies on the strategies and practices of industry formed and funded organisations, including the establishment of partnerships^25^ and the Drink Free Days initiative specifically.^14^ The critical case study method centers on empirical analysis of specific social practices and phenomena and using the findings to describe, explain and critique the dynamic relationships between contextualised cases with wider social and political regimes that maintain and legitimize particular forms of power, norms and ideas.^26^ This case study involved examining the activities of Drinkaware and the establishment of their partnership with PHE to describe, explain and critique *how* and *why* Drinkaware’s practices reproduce ways of understanding alcohol harms, what are to be seen as legitimate ways of addressing these and whose interests are served by these activities. This involved an inductive thematic analysis led by NM, supported by MvS, of written communications produced through FoI requests regarding communications between Drinkaware, PHE, and the Portman Group, in the years running up to, and during, the Drink Free Days campaign, drawing iteratively on the wider literature and publicly available documents to contextualise and explain the findings.

 The study of the activity of private firms, including their funding of other organisations, is complicated by strict intellectual property laws.^27^ As a result, researchers focused on the commercial determinants of health, and in particular, at the interface of public-private partnerships, must often rely on mechanisms such as FoI requests to access relevant data.^28^ The nature of FoI requests of this type poses a challenge for qualitative researchers because, similar to documents released during litigation, or leaks, the data represent only subset of wider communications and strategies. Due to the sensitive nature of the subject matter, these documents are often unsuitable for supplementation by in-depth interviews with, for example, those involved in these processes.^27^ To address this limitation in the context of documents released by the tobacco industry, researchers instead typically rely on triangulation across data sources, including published literature and publicly available information, to better contextualise findings from released documents.^29,30^ We therefore adopt this approach^29,31^ in parallel to our examination of the released documents, by linking to public statements and other publicly available information regarding the events under analysis covered by the documentation.

###  Data Collection

 First, two separate FoI requests were submitted under the FoI Act to PHE requesting (1) any electronic copies of correspondence and (2) documents shared, and minutes of meetings held, between the chief executive of PHE and either Drinkaware UK, the Portman Group or the International Alliance for Responsible Drinking (another alcohol industry formed and funded organisation), between January 1, 2015 and March 22, 2019. This resulted in 186 pages of documentation which formed the primary data set.

###  Analysis

 Documentary data obtained through these requests were then imported to NVivo for Mac (Release 1.6.2, QRS International, Melbourne) and read and coded iteratively by date, involved parties and key topics by NM, in accordance with the chronology of key public events regarding the campaign and events predating it (such as the launch of the revised Chief Medical Officers drinking guidelines in the United Kingdom in 2016, and the development and publication of an evidence review of alcohol policies by PHE published in December of that year). This analysis was a dynamic process, moving “back and forth” between the FoI data and the wider publicly available literature. All data was read by the research team (NM, MvS, and MP) who met regularly throughout the study to openly discuss the findings and their contextualisation.

## Results

 The results section is presented in chronological order to help contextualise the primary data, and aid in understanding the manner in which partnership working evolved over the course of the case study. This includes communications prior to the campaigns announcement, through to the campaign launch, and responses to external criticism post-launch, combining relevant quotes from primary data, and publicly available information where relevant to aid in triangulation of key events forming the case study.

###  Communications Between PHE Chief Executive and the Portman Group Before the Drink Free Days Campaign 

 Emails obtained through FoI request show that senior figures in PHE were already meeting with representatives of the alcohol industry body Portman Group prior to the commencement of the Drink Free Days campaign. On July 28, 2016, the chief executive of PHE received a letter from the Portman Group noting a meeting they described as “*…constructive and positive*” in January of that year, before going on to “*seek reassurance”* in some areas that *“…could slow progress or damage partnership working.”*

 One area of concern was a planned PHE evidence review on alcohol policy^32^ in development at the time. In a letter, the Portman Group sought to argue that “*alcohol-related harms are disproportionately concentrated in the most socio-economically deprived communities*” and asked how this would be reconciled with the PHE evidence review focusing on national policy frameworks on price, availability, and marketing. In this exchange, the Portman group letter also suggests that this was discussed in previous meetings:* “…when we met, we discussed the Evidence Review being carried out by PHE looking at the price, availability and marketing of alcohol*.” The letter also took issue with the lowering of the drinking guidelines to 14 units for men announced that year by the UK Chief Medical Officer, arguing *“This change to the guidelines resulted in an overnight reclassification of around 2.5 million male drinkers from ‘low risk’ to ‘increasing risk’ which, we believe, will only undermine efforts to convince drinkers that low risk drinking is sensible and achievable.”* The letter attempts to dispute the alcohol guidelines, justifying this by quoting media reports, criticism from the Royal Statistical Society, and what it claims are changes to mathematical modelling that underpinned the guidelines as being “…*irrational and completely fail any common response test*.”

###  Portman Group Expresses Concerns About the Makeup of PHEs Expert Advisory Panel

 Notably, the Portman group used such correspondence to take issue with the composition of the expert advisory group at PHE, quoting national press articles about its membership including those with “*ideological positions*” who had “*presented themselves as independent*.”

 Later that year, on October 3, 2016, the Portman Group again wrote to the head of PHE, referencing unreleased correspondence, expressing concern that a 21-billion-pound estimate of alcohol costs to society was out of date. The letter again raises concerns with PHE’s expert advisory group, requesting the membership be published “*as a matter of urgency*,” and “*provide assurances that your conflict-of-interest assessments prevent those with links to the Alliance House Foundation, the Institute of Alcohol Studies (IAS), the Global Alcohol Policy Alliance (GAPA), the International Organisation of Good Templars (IOGT), or any part of the temperance movement, from membership with the group*.”

 It goes further, requesting *“…reassurance that members of the group do not have track records in publicly campaigning for or against any of the alcohol policy evaluations your evidence review will be evaluating, as this would make it impossible for them to be objective or independent in their advice…*.”

 The letter also seeks to frame declines in alcohol consumption as being a consequence of corporate social responsibility activities of the industry, stating: “*I am disappointed that you refer to the very encouraging, decade-long, cultural changes around underage drinking as ‘coincidental trends,’ rather than acknowledging them to be the result of important coordinated work by public, private and voluntary sectors …the interventions currently taking place are clearly making a difference and we should be championing this approach.”*

 This aspect of the correspondence demonstrates some of the wider hidden effects that industry “responsibility” bodies like the Portman Group can exert, seeking to influence the composition of committees, including through the exclusion of civil society organisations and experts that might be detrimental to business aims. In parallel, the industry is portrayed as a pragmatic partner and source of positive trends, without evidence to support this contention, while a range of alcohol charities that do not receive industry funding are framed by the industry as extremist or “anti-alcohol.”

 The correspondence suggests that an agreement was made for further joint working: *“I would of course be happy to meet with (name withheld), as you suggest, to discuss how we can work together on a number of initiatives.”* There are also references to reciprocity, and the implication of equivalence between the work of PHE to improve health, and that of the Portman Group: *“Please be in no doubt that we are committed to reducing the harms related to alcohol misuse and will continue to support PHE’s work where we can. However, in return we expect the same level of public support from PHE for our important work at both a national and local level as we have from other parts of government.”*

###  Communications With Drinkaware and the Portman Group in Advance of the Campaign

 The correspondence reveals that high-level meetings between PHE and both Drinkaware and the Portman group occurred again in advance of the campaign being developed. The Chief Executive Officer (CEO) of Drinkaware at the time wrote *“…Drinkaware and I are very much looking forward to meeting you on Tuesday 25th July 2017 at 11.30 am….”* Later that day, following the meeting, the PHE chief executive responds: *“Thank you (name withheld) and Elaine [then-CEO of Drinkaware] for coming to meet me with (names withheld) this morning. I appreciated the frank exchange of views. We undertook to meet again in October, when we will come to the Drinkaware offices and take stock of progress. It was good to be reminded that Drinkaware’s constitutional role is educational. We agreed that there will be practical opportunities to work together and to co-badge where appropriate.”*

 Later that year, in correspondence dated October 17, 2017, the PHE chief executive appears to meet again with the Portman Group: *“Dear (name withheld), it was a pleasure meeting you today with (name withheld). We are keen to develop a relationship with the alcohol industry more akin to that we have with the food industry and look forward to furthering our discussions when you are ready for this.”*

 It appears that such work continues, as the following year, the chair of the Portman group on August 24, 2018 writes: “*We need to do much more, particularly in educating the public about the harm of excessive consumption. The need is urgent. To that end I’m hugely enthusiastic about the work PHE and PG [Portman Group] are embarking upon to develop such guidance and I was very impressed with Duncan Selbie’s (Chief executive of PHE) thoughtful contribution when he met my Board earlier this year. But progress has been a little slow, perhaps reflecting that there are some handling challenges for both of us in this enterprise. So I’d like to suggest that you and I, and Duncan and John Timothy, my CEO, meet for lunch to discuss how we can ensure this initiative succeeds.*” The head of PHE responds: “*Thank you for this and my apologies for late response with only recently returning from leave. Rest assured we are seeking ways of this being on track in the very near future and in the meantime, let’s find a time to speak*.”

 It is notable that this level of cooperation (between the Portman Group and PHE) appears to be at odds with later public assurances made about relationships between PHE and the alcohol industry, in the face of criticism of the Drink Free Days campaign when it became public. For example, the chief executive of PHE responded to criticisms of the partnership at the time in comments to the British Medical Journal explaining that “*We’re working closer with Drinkaware—that’s not the alcohol industry…. It is working with an educational charity that is charged with reducing the harm of alcohol. People are conflating Drinkaware with the alcohol industry. I think it is wrong to do that.*”^33^ It also suggests that how to communicate risk on alcohol was an area in which the industry’s views and cooperation were being actively sought.

###  Coordination Between PHE and Drinkaware on the Nature of the Campaign Itself

 The Drink Free Days campaign launched in September 2018. In the run up to the launch, in April 2018, the head of Drinkaware wrote to the head of PHE, suggesting that PHE and Drinkaware had already advanced work on this campaign: *“Since you and I last met there has, as you will know, been a great deal of work between our teams. While some real progress has been made, and Drinkaware has invested in further research in collaboration with your team, I think it’s clear that you and I need now to meet with (head of Drinkaware) and (names withheld) to determine if we do indeed have a way forward.”* A planned call then goes ahead on the 8th of May, after which the head of PHE writes: “*Thank you for our call with [sic] morning with (name withheld), I am delighted, subject to your Board’s agreement, we are to collaborate on a multi-year consumer facing campaign on promoting responsible drinking. In terms of resources PHE cannot promise cash but we do commit the full strength and reach of our One You platform and marketing expertise and we recognise you also bring the latter as well as significant moneys to underpin the joint campaign. With the increasing focus on responsible drinking we are choosing an opportune time to work in partnership together and you have our fullest commitment to making a great success of this. I look forward to joining your conference on the 22*^nd^ *.”*

 This communication offers an example of the transactional nature of the partnerships, but also conveys several of the less visible functions played by Drinkaware and similar organisations. Concepts of “*responsible drinking*” are known to be used almost exclusively in industry-funded communications, and have been found to be vague and strategically ambiguous.^9,34,35^ Through these interactions, such industry-friendly concepts, and the campaigns associated with them, appear to spread to other organisations, enabled in part by the “*pragmatism*” of deriving funding in large part from the alcohol industry, via Drinkaware. Furthermore, the campaign would ultimately focus on “*drink free days*,” rather than promoting the governments low risk drinking guidelines, which had not (and still have not) been the subject of an awareness campaign. This outcome is strategically consistent with efforts observed in past emails by the Portman Group to oppose the lowering of the guidelines. The choice of Drinkaware as partner, instead of another alcohol charity, is also strategically favourable to the Portman group, which is seeking to frame other alcohol charities who do not receive alcohol industry funding, like the IAS, as “*anti-alcohol*.”

 It is also notable that the partnership between PHE and Drinkaware appears to have been first announced at the Drinkaware conference in May 2018, a closed event, rather than to wider public health or civil society stakeholders. The day after the conference the head of Drinkaware writes to the head of PHE, stating: “*Dear Duncan, I wanted to send a personal thank you for your speech at our Conference yesterday. We were delighted you were able to deliver the keynote address and it was extremely thought-provoking. We have had very positive feedback from delegates too. I am so pleased that you were able to announce our partnership and look forward to working closely with your team.*”

###  Coordinating Efforts to Manage the Negative Response to the Drink Free Days Campaign

 The responses by PHE and Drinkaware upon the launch of the Drink Free Days campaign offer further examples of the functions served by Drinkaware and similar alcohol industry funded health information charities, and how their structure serves those functions. News of the campaign led to criticism from alcohol charities, PHE’s own advisory panel on alcohol, which did not appear to have been consulted on the campaign (consistent with the emails above, which suggest this partnership was instead agreed at the highest level within PHE), and from the Association of Directors of Public Health.

 Internal emails reveal that Drinkaware sought to push back strongly on this criticism, writing to the President of the Association of Directors of Public Health on August 27, 2018 stating: “*While it is encouraging to learn that your Association fully supports this message we are obviously concerned that the Association is nevertheless opposed to Drinkaware’s participation in the campaign on account of our industry funding.”* They go on to say they object *“in the strongest possible terms”* to any suggestion that Drinkaware is anything other than *“a totally independent body,”* and that if the Association has* “any evidence to the contrary we would be grateful to be informed of it.”* The head of Drinkaware then forwards this correspondence to the head of PHE.

 Two days later, the head of PHE writes to the Drinkaware chief executive and others and references a meeting that morning he had with the Alcohol Leadership board (a collaboration of around 50 Royal Colleges, Charities, and national and local organisations). He makes it clear the campaign is facing opposition but that he intends to both proceed, and work with Drinkaware to mitigate any negative effects: *“First to say that PHE wishes a long-term partnership with Drinkaware organization that this campaign is only the opening move. It is also fair to say that the wider public health family do not wish to see this happen or to further develop believing that Drinkaware have a poor track record of speaking to the evidence and have been slow to show independence from the industry. The feelings on this run high and they have counselled me in the strongest terms to withdraw PHE support but you should know that this is not going to happen.*” He goes on to say: *“It is inevitably of the upmost importance that we work seamlessly together in ensuring that everything we say and do can be evidenced and that our concern is solely to reduce alcohol harms. We both of course share this ambition and I am confident that we can reframe attitudes but this will be dependent on how we show that whatever was perceived of the past is consigned there as we move forwards.” *

 The next day the head of Drinkaware responds on behalf of herself and the chair of the board of trustees. *“We greatly regret the accusations which were clearly levelled at Drinkaware at your meeting yesterday. It is clear to us that perceptions of Drinkaware remain at odds with the reality of our organization despite the considerable changes we have made over the past five years… …It almost goes without saying that we agree wholeheartedly on the importance of ensuring that everything we do, together with and apart from PHE, is evidence-led. To this end, we would indeed welcome a meeting at the earliest opportunity…” *This exchange is notable in that it demonstrates the extent to which the existence of the partnership, and of the joint initiative, creates a mutual dependency that takes precedence over the concerns of such a wide range of civil society actors. This also appears at odds with a desire to be “evidence-led,” as in some cases, the weight of evidence may point to the need to terminate or prevent certain partnerships, yet this is seemingly not viewed as a viable option once the process of partnership has begun.

###  Concerns Emerge About the Nature of Health Promotion Materials Used

 The week before the campaign formally launched, concerns begin to be raised about the content on the Drinkaware website itself, in particular the level of information provided to the public on the well-established causal relationship between alcohol and cancer. In advance of a meeting arranged for 5th September, the head of PHE asks if “…*our respective medical advisors could speak before then about the cause or contribute reference to cancer. We believe the evidence says cause and that the weight of academe supports this. It is not a small matter that we get this right to those who do not wish our nascent partnership to thrive. Could I ask every effort is made to resolve this tomorrow.”*

 The reference to cancer is notable, as alcohol industry-funded organisations including Drinkaware have been found to mislead the public on the causal relationship between alcohol consumption and cancer in their materials.^3,36^ Two days later, after the meeting between the heads of PHE and Drinkaware takes place, the head of PHE summarises a number of agreed actions in an email to Drinkaware staff, including *“to address the perception on the evidence question, we agree to jointly commission a review of your website and associated materials and I suggest we engage DHSC in this too… …PHE would also be glad to join your medical advisory panel and (name withheld) was open to this… …we agreed to work together on the comms explaining why we are working together and I know this is already in hand.”* These communications give an insight into how the partnership with Drinkaware had now become an issue of reputational management for PHE, requiring allocation of additional resources. It is notable that both PHE and Drinkaware, in advance of the campaign’s launch, were already seeking to find ways to defend both the partnership and the perception of Drinkaware’s independence, in the face of criticism. It also suggests that the commitment to increasing collaboration, including PHE joining the Drinkaware medical advisory panel, could serve to further legitimize Drinkaware more broadly even as these concerns regarding accuracy surfaced.

 Of note, there was substantial reluctance on the part of Drinkaware for PHE to be seen to be correcting or amending any aspect of the Drinkaware website publicly. An unnamed account at Drinkaware responds specifically on the topic of the review of the website, saying *“…the Board will be very mindful that our website is, with some 10 million visitors a year, our single most important and unique asset… …we will need to consider very carefully the scale, nature, timescale and terms of reference of such a review and who is commissioned to carry it out… …it must be for Drinkaware to put forward proposals on these issues in the first instance and we will do so.”* They also request that *“any recommendations that emerge from the review are made formally to the Drinkaware board.” *The chief executive of PHE agrees to these requests, clarifying: *“the key thing is to address the undeniable perception, whether accurate or not, that the website is not speaking to the evidence or where it does, only partially. For example the evidence on price. This is simply a matter of securing confidence, or in more commercial language, a due diligence exercise between partners who have committed to work together.” *The reference on price is likely a reference to the effectiveness of reducing harm through reducing alcohol affordability, which alongside reducing availability, and restricting marketing, are known as alcohol policy “best buys.”

###  Efforts to Allay Criticisms Shift to Changing the Drinkaware Website

 After PHE held its annual conference that year, communications suggested that they felt criticism regarding the partnership’s initial launch had been managed, and efforts within PHE appear to shift to criticism on content by making changes to the Drinkaware website. On 13th September, a staff member (name redacted) from PHE contacts the CEO of Drinkaware to say that the PHE conference mood was “*far more positive than the minority academic view we have been handling over the last couple of days.*” They do however go on to state: “*…there is a view from the public health world that despite your website containing clear messages about alcohol harms, you don’t acknowledge the PHE alcohol evidence review as being the key summary of alcohol harm and reduction interventions. I think if you had a simple link to this on your homepage it would go some way to closing down the criticisms of these ivory tower professors*.” This references a critical aspect of such organisations, which is that industry-funded alcohol information organisations have been found to omit information on alcohol policies, or policy effectiveness, in comparison to independent charities.^6,7,9,34^ It is notable that the issue is only raised after the campaign launch, and that the proposed solution is to add a link within the Drinkaware website as a way to prevent criticism, rather than to consider how the selective omission of such information by such charities might serve a key purpose for the alcohol industry. Based on these internal emails, it appears that up until this point PHE’s concerns were focused on the correctness of information on the Drinkaware website, rather than on considering how it is framed, and what information is absent from the site altogether, such as information for readers on the evidence for minimum unit pricing, price regulation more generally, or alcohol availability, or marketing restrictions.

###  Reputational Effects for Drinkaware

 While not reported at the time, the emails also reveal the benefits for Drinkaware of the partnership itself. In a Drinkware email reporting topline results of a reputation survey of 2000+ UK adults in early December 2018 following the campaign, 32% of participants *“…felt it made their opinion of Drinkaware more positive*,” and 34% reported *“…it made Drinkaware more credible*.” Some 55% of respondents felt the partnership “*enhanced Drinkaware’s reputation*.” The campaign also led to greater traffic to the Drinkaware website. PHE’s published evaluation of the campaign states that “…*the Drinkaware site appeared at the top of search results for ‘drink free days’ for most of the campaign period meaning significant levels of traffic landed on the Drinkaware website rather than the intended Drink Free Days microsite*.”^37^

## Discussion

 This examination of internal communications before, during and after the launch of the Drink Free Days campaign shows how the campaign and partnership served wider alcohol industry objectives and complemented ongoing efforts by the Portman Group to frame alcohol harms as associated with heavy use, to define who is to be seen as a legitimate actor in addressing these harms, and to promote individual-level solutions based on education and personal responsibility as effective and sufficient. This analysis also shows that the partnership may have had other effects, such as fostering division within the public health community by marginalising those expressing critical views, developing relationships of mutual dependency between Drinkaware and PHE, maintaining active coordination to limit criticism from civil society and academics, and contributing to distracting attention from areas the industry was keen to avoid, namely a focus on evidenced-based national policy (which was lacking from the Drinkaware website and its work more generally), and on the lowering of the drinking guidelines (as the campaign in question did not focus on this, and to date, no national campaign has). Our findings explicitly show the strategic importance of such partnerships to organisations like the Portman Group and Drinkaware, including the legitimacy afforded to them through public endorsements by Government and bodies like PHE.

 Our analysis also adds to the body of evidence demonstrating the contradictions at the heart of industry discourse and evidential practices, which is observed consistently across commercial sectors.^38^ On the one hand commercial actors seek to undermine the evidence base of public health policies unfavorable to their interests using “pseudo-scientific” critique, while promoting their preferred policy measures as effective based on predominantly industry-funded reports and literature.^38^ Here we see the same phenomenon at play, whereby the Portman Group called into question the evidence base of PHE recommendations, presenting themselves as an authoritative voice capable of such critique, while promoting poorly evidenced interventions such as responsible drinking campaigns, self-regulatory arrangements or local corporate social responsibility initiatives^1,32,39-43^ as a potential response to the considerable public health threat posed by alcohol harms.

 More broadly, this study contributes to a growing body of evidence regarding the wider strategic effects of partnerships, including how they shape discourse and notions of partnership and policy priorities. A study of interactions between the US National Institute on Alcohol Abuse and Alcoholism and the alcohol industry, also involving documentation obtained via FoI request, revealed how the formulation of relationships with senior leaders in the National Institute on Alcohol Abuse and Alcoholism allowed the industry privileged access to information, involvement in substantive discussions of scientific issues, the opportunity to dispute authoritative reports and public health principles, and discuss how best to convey health information to the public.^2^ This is consistent with funding partnerships in the context of sugar sweetened beverages, that facilitated similar influence regarding policy priorities, evidence and definitions of expertise regarding obesity prevention.^44^

 The influence exerted via this partnership appeared to serve several legitimizing and delegitimising functions. These included shutting down external academic criticism of such partnerships (“…these ivory tower professors”), while offering concessions to internal partners linked to the industry. The framing by industry representatives of certain civil society actors as “anti-alcohol” or “biased” based on their advocacy for specific alcohol policies is reminiscent of similar strategies pursued by the tobacco industry in seeking to divide public health stakeholders and arrest its own delegitimisation.^45^ Similarly, efforts to create doubt regarding the process for the development of the low risk drinking guidelines are reminiscent of efforts to undermine policy-relevant research.^46^

 A greater understanding of the wider role of such partnerships, mediated through industry-funded third party organisations, may help public health researchers and practitioners seeking to examine the system-level effects of commercial activity, including in legitimizing industry-friendly evidence and practices, and delegitimising public health evidence and civil society organisations.^11,47^ It is now increasingly accepted that the commercial determinants of health represent a substantial obstacle to evidence-informed public health policy, and that empiric examination of sometimes complex and indirect pathways of influence may help to inform more effective regulatory approaches.^48^ FoI requests offer an important window into undisclosed interactions of this type.^2,28,49^

###  Limitations

 There are several limitations associated with this study methodology. It is not possible to ascertain how representative internal documentation received via responses to FoI requests may be of the wider dynamics between the organisations. It is possible that the documentation received represents only a small portion of wider interactions among a wider network of individuals and organisations. Conversely, these may represent a large portion of the interactions, though considering the implied ongoing communications and work in the emails, this may be less likely. Nevertheless, the documentation, triangulated with public statements, key dates and prior literature on the activities and purpose of such charities represent a rare illustration of the dynamics of these types of interaction, particularly as a partnership moves from design, to announcement, to execution, and then to the management of negative external perceptions. Future research could further seek to engage qualitatively with the perspectives of participants in such partnerships, to ascertain their motivations, perspectives, and reflections, as has been done with researchers who had chosen to work, or not, with the alcohol industry.^50,51^

## Conclusion

 The example of the Drink Free Days campaign suggests that the wider, system-level impacts of such organisations on policy and health are likely more profound from a public health perspective than previously assumed. Notably, such effects can include the denormalisation and marginalisation of important voices, such as other charities and health experts. Those public health stakeholders who were critical of such partnerships may be deemed unsuitable to evaluate or partner with in future initiatives. The norms surrounding conflicts of interest and partnership may as a result become shifted towards industry and away from academia and civil society. This builds on patterns seen following the Public Health Responsibility Deal in the United Kingdom, where such charities, when voicing concern about the industry-friendly direction of the initiative, found their access to policy-makers restricted compared to industry stakeholders.^52,53^ In other words, there is a denormalisation of existing partnerships with civil society in parallel to the normalization of more industry-friendly arrangements. While government agencies may view such partnerships as complementary to more evidence-based approaches, these may be intended by commercial actors to displace said approaches, directly or indirectly, and may serve to change how problems and solutions are framed in ways that undermine achieving public health goals.^54^

## Acknowledgements

 The authors would like to acknowledge constructive input on previous drafts from Rob Ralston, Jeff Collin, and Jim McCambridge.

## Ethical issues

 As documentation was in the public domain, the study did not require ethical approval. The names of staff revealed in FoI requests, except for those clearly identifiable due to being the public leaders of organisations in question, were redacted.

## Competing interests

 MP co-chaired the committee which reviewed and revised the UK Chief Medical Officer’s guidelines. The other authors declare no relevant conflicts of interest.

## Funding

 MP is a co-investigator on, and both he and NM were funded by SPECTRUM consortium which is funded by the UK Prevention Research Partnership (UKPRP), a consortium of UK funders [UKRI Research Councils: Medical Research Council (MRC), Engineering and Physical Sciences Research Council (EPSRC), Economic and Social Research Council (ESRC) and Natural Environment Research Council (NERC); Charities: British Heart Foundation, Cancer Research UK, Wellcome and The Health Foundation; Government: Scottish Government Chief Scientist Office, Health and Care Research Wales, National Institute of Health Research (NIHR) and Public Health Agency (NI)]. MvS is funded by the National Institute for Health Research (NIHR) Doctoral Fellowship (NIHR3000156) and her research is also partially supported by the NIHR Applied Research Collaboration North Thames. The views expressed are those of the authors and not necessarily those of the NIHR or the Department of Health and Social Care.
